# Single-shot compressed optical field topography

**DOI:** 10.1038/s41377-022-00935-0

**Published:** 2022-08-02

**Authors:** Haocheng Tang, Ting Men, Xianglei Liu, Yaodan Hu, Jingqin Su, Yanlei Zuo, Ping Li, Jinyang Liang, Michael C. Downer, Zhengyan Li

**Affiliations:** 1 School of Optical and Electronic Information & Wuhan National Laboratory for Optoelectronics, Huazhong University of Science and Technology, Wuhan, Hubei China; 2 Centre Énergie Matériaux Télécommunications, Institut National de la Recherche Scientifique, Université du Québec, Varennes Québec, Canada; 3 Laser Fusion Research Center, Chinese Academy of Engineering Physics, Mianyang, Sichuan China; 4 Department of Physics, University of Texas at Austin, Austin, TX USA; 5 Optics Valley Laboratory, Wuhan, Hubei China

**Keywords:** Imaging and sensing, Ultrafast photonics

## Abstract

Femtosecond lasers are powerful in studying matter’s ultrafast dynamics within femtosecond to attosecond time scales. Drawing a three-dimensional (3D) topological map of the optical field of a femtosecond laser pulse including its spatiotemporal amplitude and phase distributions, allows one to predict and understand the underlying physics of light interaction with matter, whose spatially resolved transient dielectric function experiences ultrafast evolution. However, such a task is technically challenging for two reasons: first, one has to capture in single-shot and squeeze the 3D information of an optical field profile into a two-dimensional (2D) detector; second, typical detectors are only sensitive to intensity or amplitude information rather than phase. Here we have demonstrated compressed optical field topography (COFT) drawing a 3D map for an ultrafast optical field in single-shot, by combining the coded aperture snapshot spectral imaging (CASSI) technique with a global 3D phase retrieval procedure. COFT can, in single-shot, fully characterize the spatiotemporal coupling of a femtosecond laser pulse, and live stream the light-speed propagation of an air plasma ionization front, unveiling its potential applications in ultrafast sciences.

## Introduction

Femtosecond laser pulses excite matters experiencing ultrafast evolution within femtoseconds to attoseconds^1,2^, having broad applications such as laser manufacturing, particle acceleration, and novel coherent X-ray sources^3^. The ultrafast evolution dynamics is complicated and influenced by the spatiotemporal optical field distribution of the femtosecond laser pulse, which is supposed to be completely characterized^4^. Complete characterization of a femtosecond laser pulse with a topographic “map” of the optical field enables one to understand and control ultrafast phenomena. Such a topographic map should satisfy three criteria. First, the optical field map is drawn in single-shot for ultrafast events unrepeatable or with significant shot-to-shot variations. Second, the map resolves the three-dimensional (3D) spatiotemporal profile of an optical field, i.e. two dimensions in transverse spatial directions and one dimension in the longitudinal temporal direction. Finally, the optical field map includes complete amplitude and phase information.

Capturing 3D optical field information with a two-dimensional (2D) sensor in single-shot is challenging. A quasi-3D solution is to sample a finite number of discrete 2D spectral or temporal “slices” of the 3D ultrafast scenes, such as imaging with chirped multispectral components^5–7^ or at a few specific time delays^8,9^. However, the multi-slice stack of 2D images cannot resolve complicated 3D spatiotemporal structures of an ultrafast optical field with intense amplitude or phase modulations.

Alternatively, the compressed sensing principle solves the dimensionality problem by seeking a sparse domain of an optical scene and compressing the 3D profile into the 2D detector domain^10,11^. Coded aperture snapshot spectral imaging (CASSI) can measure the 3D spectral intensity profile $\left| {E\left( {x,y,\omega } \right)} \right|^2$ of an optical field in single-shot^12^, by spatially modulating the intensity profile with a randomly patterned binary coded aperture, and shearing frequency-dependent intensity profiles on the 2D detector with an angular dispersive optics such as a prism. Compressive sensing based algorithms are applied to reconstruct 3D spectral intensity profile $\left| {E\left( {x,y,\omega } \right)} \right|^2$ from a 2D image captured by the detector in a single shot (details in Methods)^13^. Based on a similar principle, compressed ultrafast photography (CUP)^14,15^ visualizes a 3D spatiotemporal intensity profile of an ultrafast optical field by replacing the angular dispersive optics with an ultrafast streak camera. Compressed ultrafast spectral-temporal photography (CUSTP)^16,17^ also provides an ultrafast frame rate using a chirped pulse with a linear frequency-time mapping relation. Generally, compressed sensing based ultrafast photographic techniques draw a 3D “intensity” map of an optical “field”, leaving phase information unresolved.

Now the 3D phase profile is still in need for a complete topographic map of an optical field, requiring a single-shot global 3D phase retrieval procedure. Transverse 2D spatial phase retrieval recovers the wavefront of an optical field and constructs the foundation of coherent diffractive imaging^18–20^, even with broadband coherent light sources^21^. However, the spectral phase linking different spectral components of the ultrafast optical field is absent. Multiple ultrafast pulse measurement techniques such as frequency-resolved optical gating (FROG) can measure spectral phase in single-shot^22^, however, it assumes uniform transverse spatial distribution^23,24^. Spectral interferometry or holography using an imaging spectrometer can also measure spectral phase and ultrafast dynamics in single-shot^25–28^, however, the spectrometer slit excludes information along the transverse direction perpendicular to it. 3D phase profile measurement has been achieved by CUP complemented with a dark-field imaging scheme^29^, which converts the 3D phase profile to intensity but loses the amplitude and absolute phase value information.

Here we demonstrate compressed optical field topography (COFT) to draw a 3D topographic “map” for an arbitrary ultrafast optical field in single-shot. Based on CASSI, COFT solves the problem of 3D information capture. Two global 3D phase retrieval procedures are proposed, one is based on wavefront recovery and non-collinear FROG, and the other is based on 3D spectral holography. Both implementations succeed in visualizing the optical field of femtosecond laser pulses with spatiotemporal coupling, and the former one is applied to study the light-speed propagation of a plasma channel ionization front, illustrating the versatility of COFT.

## Results

### COFT based on wavefront recovery and non-collinear FROG

**Fig. 1:**
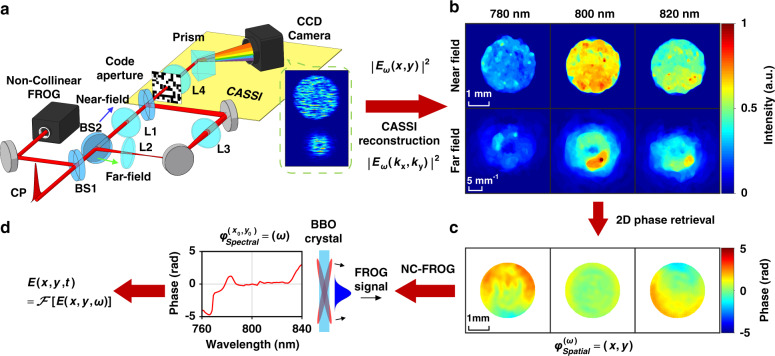
Principle of COFT based on wavefront recovery and non-collinear FROG. **a** The experimental setup. An arbitrary optical field at CP, the checking point, is split by beam splitters (BS1, BS2) into three copies: the first copy goes to the Non-Collinear FROG for spectral phase $\varphi _{{{{\mathrm{Spectral}}}}}^{\left( {x_0,y_0} \right)}\left( \omega \right)$ measurement; the second one is imaged by a lens L1 to the coded aperture; and the third one is focused by L2 to a far-field which is imaged to the coded aperture by L3. The last two copies are spatially off-set and captured by a CASSI system including the coded aperture, a lens L4, a prism, and a camera. Raw data is shown in the inset. **b** CASSI reconstruction results for the second copy corresponding to the near field intensity distribution $\left| {E_\omega \left( {x,y} \right)} \right|^2$ (the top row) and the third one corresponding to the far-field intensity distribution $\left| {E_\omega \left( {k_x,k_y} \right)} \right|^2$ (the bottom row), for three wavelengths at 780 nm (left), 800 nm (middle), 820 nm (right). **c** Wavefronts [2D spatial phase distributions $\varphi _{{{{\mathrm{Spatial}}}}}^{\left( \omega \right)}\left( {x,y} \right)$] for three spectral components with the wavelength of 780 nm (left), 800 nm (middle), 820 nm (right), which are retrieved based on corresponding near- and far-field intensity profiles shown in **b**. **d** Spectral phase $\varphi _{{{{\mathrm{Spectral}}}}}^{\left( {x_0,y_0} \right)}(\omega )$ reconstructed from the non-collinear FROG trace, with the assistance of 2D optical field information of each spectral component. The inset schematically shows the principle of non-collinear FROG: two copies of the incident pulse cross each other at a thin nonlinear BBO crystal, and they have a linearly varying temporal delay along the transverse direction, generating transversely distributed sum frequency signal or the FROG trace

The COFT system based on wavefront recovery and non-collinear FROG is shown in Fig. 1a. An arbitrary ultrafast optical field originating from the checking point (CP) is split by a beam splitter (BS1). The reflected beam enters a non-collinear FROG for spectral phase measurement, which will be discussed later. The transmitted part is split by another beam splitter (BS2). On the transmission near-field path (labeled by a blue arrow), a lens L1 images CP to the coded aperture of a CASSI system, and the optical field’s spectral-spatial amplitude profile at the coded aperture is equivalent to $\left| {E_\omega \left( {x,y} \right)} \right|$ with a magnification determined by L1. On the reflection far-field path (labeled by a green arrow), the lens L2 located after CP by its focal length takes a Fourier transform of the spatial profile of the optical field^30^, yielding the far-field amplitude profile $\left| {E_\omega \left( {k_x,k_y} \right)} \right|$ which is relayed to the coded aperture by the lens L3. The 3D hyperspectral amplitude profiles of the optical field at near- and far-fields are spatially separated and simultaneously measured by a CASSI system [Fig. 1a inset], including the coded aperture (a chromium-coated fused silica plate with a pseudo-random binary pattern), a lens L4 imaging the coded aperture to the detector, a prism introducing spectral shearing, and a charge coupled device (CCD) camera as the 2D detector. The 3D hyperspectral near- and far-field amplitude profiles $\left| {E_\omega \left( {x,y} \right)} \right|$ and $\left| {E_\omega \left( {k_x,k_y} \right)} \right|$ are reconstructed based on the CASSI principle, using the plug-and-play alternating direction method of multipliers (PnP-ADMM) technique (see Methods and Supplementary Materials)^31^. Figure 1b shows the reconstructed near- and far-field intensity profiles of the optical field for selected wavelengths and corresponding spectral components.

Global 3D phase retrieval of the optical field takes two steps. The first step recovers 2D spatial phase profiles or wavefronts for all independent frequency components $\varphi _{{{{\mathrm{Spatial}}}}}^{\left( \omega \right)}\left( {x,y} \right)$ and the second step measures the spectral phase $\varphi _{{{{\mathrm{Spectral}}}}}^{\left( {x_0,y_0} \right)}\left( \omega \right)$ of the optical field at a specific spatial position ($x_0$, $y_0$), where $\varphi _{{{{\mathrm{Spatial}}}}}^{(\omega )}\left( {x_0,y_0} \right)$ is set to be zero. Thus, the 3D phase profile is

1 $$\varphi \left( {x,y,\omega } \right) = \varphi _{{{{\mathrm{Spatial}}}}}^{\left( \omega \right)}\left( {x,y} \right) + \varphi _{{{{\mathrm{Spectral}}}}}^{\left( {x_0,y_0} \right)}\left( \omega \right)$$

By applying the Gerchberg-Saxton phase retrieval algorithm^32^ on reconstructed amplitude profiles of $\left| {E_\omega \left( {x,y} \right)} \right|$ and $\left| {E_\omega \left( {k_x,k_y} \right)} \right|$ at the near- and far-fields respectively, one can complete the first step of phase retrieval, obtaining spatial phase profiles $\varphi _{{{{\mathrm{Spatial}}}}}^{\left( \omega \right)}\left( {x,y} \right)$ for all independent frequency components [Fig. 1c].

The spectral phase $\varphi _{{{{\mathrm{Spectral}}}}}^{\left( {x_0,y_0} \right)}\left( \omega \right)$ is measured by a non-collinear FROG, in which two copies of the incident laser pulse cross each other at a thin BBO crystal plate and generate transversely distributed sum-frequency signal in single-shot [Fig. 1d]. Conventionally a non-collinear FROG measures spectral phase in single-shot based on the assumption of uniform transverse spatial distribution, COFT does not rely on this assumption and takes advantage of complete information about optical field spatial profiles $E_\omega \left( {x,y} \right) = \left| {E_\omega \left( {x,y} \right)} \right|\exp \left[ {i\varphi _{{{{\mathrm{Spatial}}}}}^{\left( \omega \right)}\left( {x,y} \right)} \right]$ for all frequency components. So instead of using a standard complex projection FROG phase retrieval algorithm^22^, an evolutionary algorithm is developed to reconstruct the one-dimensional spectral phase $\varphi _{{{{\mathrm{Spectral}}}}}^{\left( {x_0,y_0} \right)}\left( \omega \right)$ from the measured FROG trace (see Supplementary Materials)^33^. Once $\varphi _{{{{\mathrm{Spatial}}}}}^{\left( \omega \right)}\left( {x,y} \right)$ and $\varphi _{{{{\mathrm{Spectral}}}}}^{\left( {x_0,y_0} \right)}\left( \omega \right)$ are both reconstructed, the global 3D phase profile $\varphi \left( {x,y,\omega } \right)$ is obtained [Eq. (1)], and one-dimensional Fourier transformation of $E\left( {x,y,\omega } \right) = \left| {E_\omega \left( {x,y} \right)} \right|e^{i\varphi \left( {x,y,\omega } \right)}$ yields the spatiotemporal profile of the optical field $E\left( {x,y,t} \right)$.

We first use COFT to measure the optical field of a femtosecond laser pulse. As large-scale petawatt femtosecond laser facilities are constructed worldwide^34^, advanced single-shot characterization techniques are required^35^ to resolve spatiotemporal coupling (STC)^36^ at a low repetition rate. Previously, multi-shot techniques scanning spatial positions^37^ or temporal delays^38–40^ can measure the optical field of femtosecond laser pulses with negligible pulse-to-pulse variations. Single-shot techniques “multi-spectrally” measure spatial profiles of an optical field in a few wavelength channels^5,6,41^. However, as far as we know, drawing a real 3D map in a single shot (not a series of 2D slices) for the optical field of a femtosecond laser pulse is still challenging.

**Fig. 2:**
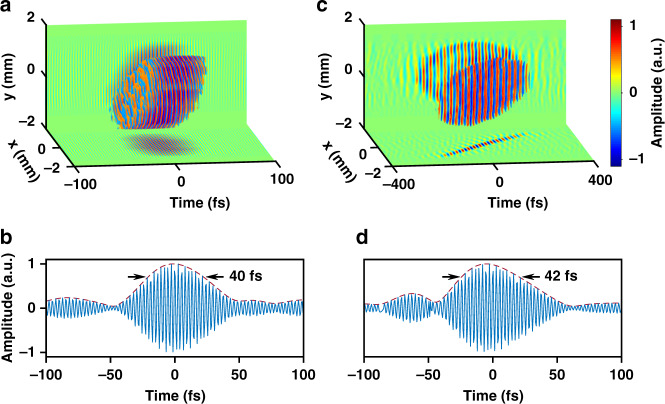
3D topographic maps of femtosecond laser pulses measured by COFT based on wavefront retrieval and non-collinear FROG. **a** Spatiotemporal distribution of the electric field of a femtosecond laser pulse from a kilohertz, millijoule femtosecond laser amplifier. **b** Temporal profile of the electric field (blue lines) at the central point $\left( {x,y} \right) = \left( {0,0} \right)$ of the laser pulse in **a**, and the red dashed line shows the amplitude envelope. **c** Similar to **a**, but for spatiotemporal electric field distribution of a femtosecond laser pulse with spatiotemporal coupling, which is induced by propagating the laser pulse in a glass prism at CP. To avoid too fast oscillations of the field and get a clear visualization, the carrier wave frequency in **c** has been numerically reduced by 10 times. **d** Similar to **b**, but for the temporal profile of the electric field at the central point in **c**. To clearly show the peaks and valleys of the optical fields in **a** and **c**, only regions where the absolute amplitude is higher than 0.4 times the peak absolute amplitude are shown

In our prototype experiments, we first measured the 3D optical field profile of laser pulses from a 1 kHz femtosecond laser amplifier (central wavelength 800 nm, pulse duration 40 fs, pulse energy up to 5 mJ). The single-shot measurement is achieved by synchronizing the CCD camera of the CASSI system with the masterclock of the laser system with a reduced trigger frequency, and controlling the camera exposure time to guarantee that only a single shot is captured in one measurement. As shown in Fig. 2a, the optical field distribution shows a clipped transverse spatial beam profile, with a spatial resolution estimated to be 50 μm determined by the coded aperture pixel size and magnification of the system. The clear edge corresponds to a 3 mm-diameter hard aperture placed at CP. Along the longitudinal temporal dimension, the pulse duration is 40 fs [Fig. 2b], and the temporal grid size 3.9 fs determines the temporal resolution, equivalent to an imaging frame rate of 256 trillion frames per second. It is also notable that the optical field distribution of the laser pulse is spatially inhomogeneous (the leading edge of the pulse is located in the area of y < 0 and the trailing edge of the pulse in the area of y > 0), justifying the necessity of single-shot 3D optical field visualization.

We have further challenged the COFT technique by intentionally introducing complicated spatiotemporal coupling to the incident laser pulse. A dispersion prism (PS856, Thorlabs) is placed at CP and introduces angular dispersion along the x-direction to the laser pulse, leading to a tilted pulse amplitude front (pulse-front tilt ~186 fs/mm) but a flat wave phase front according to the theory^42^. Figure 2c has successfully corroborated the theoretical prediction. The projection of the optical field on the x-t plane shows a clear linear tilting with a measured pulse-front tilt of 178 fs/mm, whereas all phase contours are flat and parallel to the transverse x-y plane. The temporal profile of the reconstructed optical field at x = 0 and y = 0 is lined out [Fig. 2d], the pulse duration is 42 fs, close to the undispersed pulse duration. This observation is consistent with the theory that pure angular dispersion without further propagation does not broaden local pulse duration.

Thus COFT is potentially able to characterize complicated femtosecond laser pulses for different applications. For example, one can expect the application of COFT to single-shot measurement of the spatiotemporal optical field profile of a low-repetition rate, large-aperture petawatt laser pulse, if an appropriate optical system is designed and pre-calibrated to shrink the beam size. COFT can also in principle be able to characterize more complicated optical field such as an optical vortex by measuring the 3D spectral hologram due to interference between the optical vortex pulse and a reference pulse which has no optical angular momentum and is well calibrated by COFT. In addition, the optical setup of COFT is not more complicated than other standard pulse characterization techniques, requiring neither optical elements sensitive to mechanical vibrations and misalignment nor moving parts. So COFT is robust and the measurement error due to misalignment is negligible.

COFT can characterize not only a laser pulse but also a modulated probe pulse in a pump-probe experiment, in which the 3D dielectric function profile of the pump excited matter is encoded in the 3D optical field profile of the probe. Compared to CUP and CUSTP which are applied in pump-probe experiments for intensity or amplitude modulation information of the probe, the phase measurement capability of COFT enables ultrafast photography of evolving refractive index structures.

**Fig. 3:**
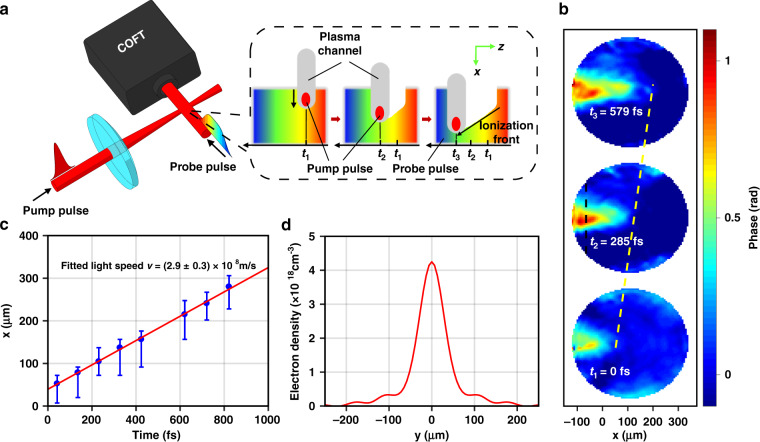
Visualization of light-speed propagation of an air plasma ionization front by COFT measurement of the probe pulse. **a** Schematic diagram of the pump-probe experiment visualizing the light-speed propagation of the air plasma ionization front in air. The pump pulse (1 mJ, 40 fs) propagating along the x-direction is focused and ionizes the air. The probe pulse detects the pump-induced refractive index shift transversely and enters a COFT system based on wavefront retrieval and non-collinear FROG, with the interacting region as CP. The inset cartoon shows the process of the probe picking up the pump-induced phase shift. At time *t*_1_, the pump and the plasma channel overlap with the leading edge of the probe; at time *t*_2_, with the probe central part; at time *t*_3_, with the trailing edge. **b** Representative frames of the probe pulse transverse phase shift profiles in the x*-*y plane at $t = 0,285,579$ fs. The yellow dashed line shows the position of the ionization front at different time. **c** Linear fitting of the ionization front positions at different time, and the slope is consistent with light speed. **d** Free electron density profile along the black dashed line in **b**, reconstructed via a standard Abel inversion scheme

To prove the idea, we have visualized the optical field of a laser pulse transversely probing an air plasma ionization front excited by an intense pump laser pulse [Fig. 3a]. The pump laser pulse (pulse energy ~1 mJ, pulse duration ~40 fs) is focused into a 75-µm diameter focal spot by a 250-mm-focal length lens, reaching a peak intensity of 6.5 × 10^14^ W/cm^2^, generating light-speed ionization front leading a plasma channel and co-propagating with the pump pulse. A probe laser pulse propagates transversely through the plasma channel and is modulated primarily in phase by the refractive index changes of the plasma channel. By setting the crossing of the pump and probe beams as CP, the map of the linearly chirped probe pulse is drawn by the COFT system with and without the pump pulse, yielding a time-resolved phase shift profile ${\Delta}\varphi \left( {x,y,t} \right)$.

Figure 3b shows the time-resolved phase map induced by the pump pulse, with the ionization front propagating towards the positive x-direction. The ionization front locates at x = 52 μm at t = 0 fs and moves to x = 137 μm and 215 μm when t = 285 and 579 fs respectively. A linear fitting between the ionization front position and propagation time shows a fixed propagation speed of (2.9 ± 0.3) × 10^8^ m/s, consistent with light speed [Fig. 3c]. The absolute phase shift values in Fig. 3b reveal plasma density. By assuming a cylindrically symmetric plasma spatial profile and applying a standard Abel inversion scheme, the maximum free electron density in the ~80 μm thick plasma channel is estimated to be 4.2 × 10^18^ cm^−3^ [Fig. 3d], consistent with previous experimental results^43^. We have also obtained amplitude modulation information of the probe, however it is small because the underdense air plasma has little absorption to the probe pulse. It is believed that COFT can also visualize evolution dynamics of, for example, plasma wakes in a laser wakefield accelerator^44^, but compared to previous techniques^45–48^, such visualization is single-shot and suitable to experiments with significant shot-to-shot variations.

### COFT based on 3D spectral holography

**Fig. 4:**
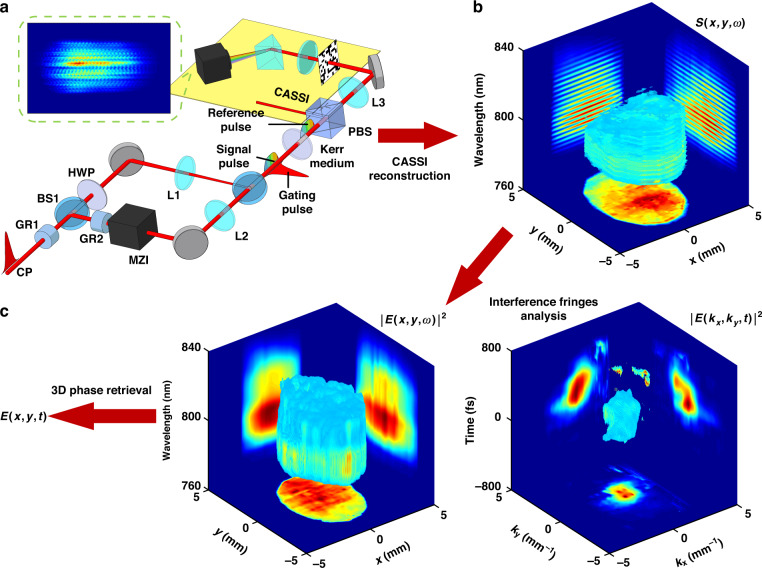
Principle of COFT based on 3D spectral holography. **a** The experimental setup. The incident optical field at CP, the checking point, is pre-chirped by an SF11 glass rod (GR1) and split by beam splitter BS1. The transmitted gating pulse is focused by the lens L1 to a thin glass plate as the Kerr medium. The reflected pulse is further chirped by an SF11 glass rod (GR2) and manipulated by a Mach-Zehnder interferometer (MZI) to generate a time-delayed reference-signal pulse pair. The reference-signal pulses are imaged by lens L2 to the Kerr medium, and the signal pulse picks up phase shift induced by the gating in the Kerr medium. After the cross-polarized gating pulse is filtered out by a polarization beam splitter (PBS), the remaining reference and signal pulses interfere and generate a 3D spectral hologram, which is imaged by lens L3 to the coded aperture of a CASSI system. Raw data is shown in the inset. **b** CASSI reconstruction of the 3D spectral hologram $S\left( {x,y,\omega } \right)$ due to interference between the reference and the modulated signal probe. **c** The $\left| {E\left( {k_x,k_y,t} \right)} \right|^2$ and $\left| {E\left( {x,y,\omega } \right)} \right|^2$ 3D intensity profiles of the incident optical field. The former is predominantly obtained from the phase shift $\theta \left( {x,y,\omega } \right)$ coded in the 3D spectral hologram. $\left| {E\left( {x,y,\omega } \right)} \right|^2$ and $\theta \left( {x,y,\omega } \right)$ are reconstructed from the 3D spectral hologram $S\left( {x,y,\omega } \right)$ through the standard interference fringe analysis procedure

The global 3D phase retrieval can also be realized based on 3D spectral holography. Figure 4a shows the schematic diagram of a COFT system based on 3D spectral holographic phase retrieval. An incident laser field at CP is pre-chirped by an SF11 glass rod (GR1) and split by a beam splitter BS1. After rotating the polarization by 90° by a half wave plate (HWP), the transmitted intense “gating” beam is focused by a lens L1 to a thin glass plate as a nonlinear Kerr medium, introducing nonlinear refractive index changes proportional to the far-field intensity profile $\left| {E\left( {k_x,k_y,t} \right)} \right|^2$. The reflected beam from BS1 is further chirped by another SF11 glass rod (GR2), and two weak, equivalent, collinearly propagating pulses with a fixed time delay *τ* are generated by a Mach-Zehnder interferometer (MZI). The leading and trailing ones of the pulse pair are named “reference” and “signal” respectively, and a lens L2 images them from CP to the Kerr medium. Thus the spectral intensity profile of the reference pulse at the Kerr medium is $\left| {E\left( {x,y,\omega } \right)} \right|^2$. The signal pulse temporally overlaps with the gating pulse at the Kerr medium, and picks up the gating-induced 3D phase shift through cross phase modulation. After filtering out the gating pulse by a cubic polarizer (PBS), the reference and the modulated signal pulses are relay imaged by L3 to the coded aperture of a CASSI system.

In the CASSI system, the reference-signal interference introduces a 3D spectral hologram

2 $$\begin{array}{ll}S\left( {x,y,\omega } \right) =\!\!\!\!& \left| {E\left( {x,y,\omega } \right)} \right|^2 \, + \, \left| {E_1\left( {x,y,\omega } \right)} \right|^2\\ &+ \,2\left| {E\left( {x,y,\omega } \right)E_1\left( {x,y,\omega } \right)} \right|\cos \left[ {\theta \left( {x,y,\omega } \right) + \omega \tau } \right]\end{array}$$

where $E_1\left( {x,y,\omega } \right)$ and $\theta \left( {x,y,\omega } \right)$ are amplitude and phase modulation of the signal pulse due to the intense gating pulse at the Kerr medium, respectively. The 3D hologram is spatially coded, sheared by the prism, captured in single-shot by the camera, and finally reconstructed using the PnP-ADMM algorithm for CASSI reconstruction [Fig. 4b]. An interference fringes analysis scheme can resolve the reference hyperspectral intensity profile $\left| {E\left( {x,y,\omega } \right)} \right|^2$ [Fig. 4c, right panel] and the 3D phase shift profile $\theta \left( {x,y,\omega } \right)$ of the signal pulse^49^.

We next link the phase shift $\theta \left( {x,y,\omega } \right)$ of the signal pulse with the intensity profile of the gating pulse at far field $\left| {E\left( {k_x,k_y,t} \right)} \right|^2$. For the transverse spatial directions, the transverse position *x* or *y* is proportional to the corresponding transverse component of the incident optical field wave vector *k*_*x*_ or *k*_*y*_, by $x = ak_x,y = ak_y$ where $a = \lambda F_{{{{\mathrm{L}}}}1}/2\pi$ and *F*_L1_ is the focal length of lens L1. For the longitudinal temporal direction, the signal pulse is chirped by GR1 and GR2, so the temporal delay *t* relative to the signal pulse and the modulated frequency component *ω* have a linear time-frequency mapping relation $\omega = t/b$^26^, where *b* is the chirp rate of the signal pulse and can be determined self-consistently using the dispersion properties of GR1 and GR2 (details in Supplementary Materials). In addition, Ref. ^26^. has explained that the linear time-frequency mapping procedure is available only when the temporal duration of the phase shift profile or the gating pulse is longer than a temporal resolution determined by the bandwidth and the chirp of the signal pulse, so the gating pulse is pre-chirped by GR1. After determining the parameter *a* and the chirp rate *b*, the 3D phase shift profile of the signal pulse $\theta \left( {x,y,\omega } \right) = \theta \left( {x = ak_x,y = ak_y,\omega = t/b} \right)$ is expressed in the $\left( {k_x,k_y,t} \right)$ domain, yielding the normalized far-field intensity profile of the gating pulse $\left| {E\left( {k_x,k_y,t} \right)} \right|^2$ [Fig. 4c, left panel]. Considering that $\left( {x,y,\omega } \right)$ and $\left( {k_x,k_y,t} \right)$ are Fourier-conjugate variables, the 3D spectral phase of the incident optical field $\varphi \left( {x,y,\omega } \right)$ and the 3D optical field distribution are obtained from intensity profiles $\left| {E\left( {k_x,k_y,t} \right)} \right|^2$ and $\left| {E\left( {x,y,\omega } \right)} \right|^2$ through a 3D phase retrieval scheme based on the Gerchberg-Saxton algorithm^32^.

**Fig. 5:**
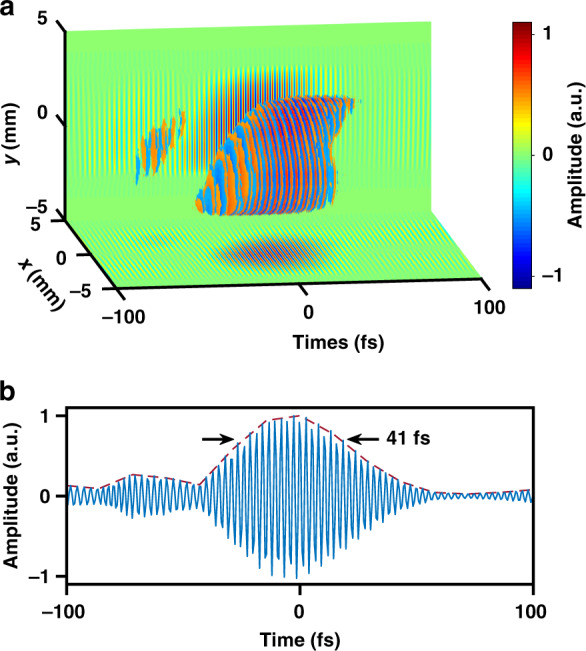
3D topographic maps of femtosecond laser pulses measured by COFT based on 3D spectral holography. **a** Spatiotemporal distribution of the electric field of a femtosecond laser pulse from the same laser system as Fig. 2. To clearly show the peaks and valleys of the optical fields, only regions where the absolute amplitude is higher than 0.4 times the peak absolute amplitude are shown. **b** Temporal profile of the electric field (blue lines) at the central point $\left( {x,y} \right) = \left( {0,0} \right)$ of the laser pulse in **a**, and the red dashed line shows the amplitude envelope

Figure 5a shows the 3D optical field profile of a laser pulse using the COFT system based on 3D spectral holography, compared to the results in Fig. 2a. Despite the difference in implementing 3D phase retrieval, both measurements show that the optical field of laser pulses from our laser amplifier has a similar spatiotemporally coupled distribution that the leading edge is at the region of y < 0 and the trailing edge at the region of y > 0. The measured pulse front tilting along the y-direction in Fig. 5a is +14.7 fs/mm, close to that of +16.5 fs/mm in Fig. 2a. In addition, a lineout of the optical field at the center of the beam in Fig. 5a gives a pulse duration of 41 fs [Fig. 5b], also close to that of 40 fs in Fig. 2b. Considering that the small pulse front tiltings intrinsic to the laser system are quantitatively measured both in Figs. 2a, b and 5 using different COFT implementations, the consistencies in results justify the robustness and repeatability of both COFT implementations.

## Discussion

We have demonstrated two implementations of the COFT technique to draw a single-shot topographic map of an ultrafast optical field. Both implementations involve combinations of CASSI and 3D phase retrieval, and take advantage of a transient nonlinear optical process as the “temporal gating function” to measure the spectral phase and the temporal profile of the pulse. For the first COFT implementation based on wavefront recovery and non-collinear FROG, the nonlinear optical gating process of sum-frequency generation is efficient and background-free, but at the price of a relatively complicated optical setup measuring spatial and spectral phase profiles separately. For the second COFT implementation based on 3D spectral holography, the 3D phase profile is retrieved from the fringe shift of the 3D spectral hologram, which is induced by the nonlinear optical gating process of cross phase modulation. This implementation has a simplified experimental setup, but the fringe analysis procedure is sensitive to the incident laser intensity. One can expect other novel 3D phase retrieval and COFT implementations in future, and we believe any of them can further promote ultrafast imaging or photography techniques in three aspects.

First, COFT can be applied to draw topographic maps of optical fields for both pump and probe pulses in a pump-probe experiment. With complete 3D amplitude and phase information of the pump obtained in single-shot, COFT allows one to predict how the pump pulse with probably a complicated spatiotemporal structure influences light-matter interaction dynamics in real experiments. Such predictions can then be verified by looking up the optical field profile of the modulated probe pulse for spatially and temporally resolved dielectric function information. For example, during femtosecond laser ablation and processing of solid materials, the pump induced complex dielectric function modulation of the material introduces both amplitude and phase modulations on the probe pulse which could be measured in-situ by COFT, implying COFT of the probe as a potential laser manufacturing metrology tool.

Second, COFT incorporating 3D phase information has an imaging frame rate improved by orders of magnitude. Previous optical compressed imaging techniques have an imaging frame rate limited to tens of trillions of frames per second, determined by the temporal resolution of a streak camera (~100 fs at minimum) for CUP^15^, and by the spectral bandwidth and the chirp applied to stretch the pulse for CUSTP^26^. COFT is based on hyperspectral compressed imaging and is supposed to have a similar temporal resolution to CUSTP. However, once the 3D phase profile especially the spectral phase is recovered in COFT, the theoretical limit of temporal resolution for the pure intensity imaging case is relaxed. For example, COFT based on wavefront recovery and non-collinear FROG can resolve spectral phase, and the minimum temporal resolution is only limited by the geometric smearing and at the order of sub-femtoseconds^22^, corresponding to a multi-quadrillion frame rate.

Third, in both implementations of COFT, optical compressed imaging is combined with a global 3D phase retrieval procedure. It is well known that iterative phase retrieval manifests itself in wavefront measurement and coherent diffractive imaging using short-wavelength coherent light sources, such as extreme ultraviolet and x-ray. Thus, it is likely to extend COFT to these novel short-wavelength spectral ranges if two main problems could be solved. First, to implement CASSI in this spectral range, appropriate imaging optics such as toroidal or Kirkpatrick-Baez mirrors should image the coded aperture to the detector with well pre-calibrated optical aberrations. Second, to obtain the spectral phase or temporal profile of a short-wavelength optical field, an efficient nonlinear optical gating process at extreme ultraviolet or x-ray spectral range should be investigated^50–52^. Once both problems are solved, short wavelength COFT is expected to visualize complete optical field information for x-ray free electron laser pulses^53^ and attosecond light bursts based on high harmonic generation^2^, and in turn applied to studies of ultrafast dynamics within sub-nanometer spatial and attosecond temporal scales in pump-probe experiments^19^.

## Materials and methods

### Principle of coded aperture snapshot spectral imaging

CASSI is to reconstruct a 3D datacube from a 2D encoded snapshot with the aid of compressed sensing based algorithms^54^. A CASSI experiment can be completed in two steps: data acquisition and image reconstruction. The process of data acquisition generally consists of three successive operations. First of all, the optical field $f\left( {x,y,\omega } \right)$ is spatially encoded by the pseudo-random binary mask, which is referred to as spatial encoding denoted by **C**. Then, the dispersive prism exerts spectral shearing (denoted by **S**) to the optical field in the horizontal direction. In the end, the signal experiences spectral integration (denoted by **I**) on a two-dimensional array detector such as a charge-coupled device. In this way, the compact form of the data acquisition can be expressed as $A\left( {x,y} \right) = {{{\mathbf{O}}}}f\left( {x,y,\omega } \right)$, where **O** = **ISC**.

Here, COFT uses the plug-and-play alternating direction method of multipliers (PnP-ADMM)^30^ framework for image reconstruction. The main idea for this algorithm is that taking the advantage of the modular structure of ADMM algorithm, a widely used algorithm for solving constrained optimization problems, any off-the-shelf image denoising algorithm can be plugged in to solve a subproblem in ADMM. To solve the under-sampled inverse problem, the optimization object is written as

3 $$\hat f = {{{\mathrm{argmin}}}}\frac{1}{2}\left\| {A - {{{\mathbf{O}}}}f} \right\|_2^2 + R\left( f \right) + I_ + \left( f \right)$$

where $\left\| \cdot \right\|_2$ is the *l*_2_ norm. The term $\frac{1}{2}\left\| {A - {{{\mathbf{O}}}}f} \right\|_2^2$ evaluates the distance between the measurement and the corresponding value from the estimated results. $R\left( \cdot \right)$ is an implicit regularizer. $I_ + \left( \cdot \right)$ represents a non-negative intensity constraint, which is a naturally satisfied physical constraint. PnP-ADMM implements a variable splitting strategy to obtain the solution, with details for each step of the algorithm shown in Supplementary materials.

The CASSI measurement system includes one static pseudo-random binary transmissive mask (a chromium-coated fused silica plate with 30 μm × 30 μm encoding pixel’s size), a 150 mm-focal-lengths imaging lens (L4), a dispersive prism (PS850, Thorlabs, with angular dispersion of 14.3 nm/mrad@800 nm), and a CCD camera (HIKROBT, MV-CA023-10UM, 5.86 μm pixel size). In experiments, the imaging magnification from the coded mask to the camera is 2, the prism is inserted 20 cm in front of the camera, and a 2 × 2 pixel binning is implemented on the camera.

## Supplementary information

Supplemental material
