# Supplementary material for: Single-shot compressed optical field topography
Source: Light Sci Appl. 2022 Aug 2;11:244. doi: 10.1038/s41377-022-00935-0 (PMC9343635; doi:10.1038/s41377-022-00935-0)
Supplement: Supplementary file 1 — Supplemental material [file 41377_2022_935_MOESM1_ESM.docx]

**Supplementary Information for**

**Single-shot Compressed Optical Field Topography**

Haocheng Tang^1*^, Ting Men^1*^, Xianglei Liu^2^, Yaodan Hu^1^, Jingqin Su^3^, Yanlei Zuo^3^, Ping Li^3^, Jinyang Liang^2^, Michael C. Downer^4^, Zhengyan Li^1,5#^

1. School of Optical and Electronic Information & Wuhan National Laboratory for Optoelectronics, Huazhong University of Science and Technology, Wuhan, Hubei, China
2. Centre Énergie Matériaux Télécommunications, Institut National de la Recherche Scientifique, Université du Québec, Varennes, Québec, Canada
3. Laser Fusion Research Center, Chinese Academy of Engineering Physics, Mianyang, Sichuan, China
4. Department of Physics, University of Texas at Austin, Austin, Texas, USA
5. Optics Valley Laboratory, Wuhan, Hubei, China

* Contribute equally

# Corresponding email: zhengyanli@hust.edu.cn

1. **Hyperspectral intensity profile reconstruction using coded aperture snapshot spectral imaging (CASSI)**

COFT uses the plug-and-play alternating direction method of multipliers (PnP-ADMM) technique to reconstruct the hyperspectral intensity profiles $f\left( x,y,\omega\right)$ from the measured signal $A\left( x,y \right)$, where $A=\mathbf{O}f\boldsymbol{=}\mathbf{ISC}f$. Mathematically this is an inverse problem with under-sampled measurements, and the idea of ADMM is to convert the unconstrained optimization problem described by Eq. (2) in the main text into a constrained optimization problem

$\begin{aligned} \hat{f}={\mathrm{argmin}\frac{1}{2}\left\| A-\mathbf{I}u \right\|}_{2}^{2}+R\left( v \right)+I_{+}\left( w \right)& \\ s. t. u=\mathbf{SC}f,v=f,w=f \end{aligned}$ (S1)

and consider its augmented Lagrangian function:

$\begin{aligned} L\left( f,u,v,w \right)&=\frac{1}{2}\left\| A-\mathbf{I}u \right\|_{2}^{2}+R\left( v \right)+I_{+}\left( w \right) \\ &+\frac{\mu_{1}}{2}\left\| \mathbf{SC}f-u+\frac{\gamma_{1}}{\mu_{1}} \right\|_{2}^{2}+\frac{\mu_{2}}{2}\left\| f-v+\frac{\gamma_{2}}{\mu_{2}} \right\|_{2}^{2}+\frac{\mu_{3}}{2}\left\| f-w+\frac{\gamma_{3}}{\mu_{3}} \right\|_{2}^{2} \end{aligned}$ (S2)

Here, $\mu_{i}(i=1,2,3)$ are penalty parameters, and $\gamma_{i}(i=1,2,3)$ are dual variables. $u,v,w$ are functions updated in every iteration by solving sub-problems below:

$u^{(k+1)}=\mathrm{argmin} \frac{1}{2}\left\| A-\mathbf{I}u^{(k)} \right\|_{2}^{2}+\frac{\mu_{1}}{2}\left\| \mathbf{SC}f^{\left( k \right)}-u^{\left( k \right)}+\frac{\gamma_{1}}{\mu_{1}} \right\|_{2}^{2}$ (S3)

$v^{\left( k+1 \right)}=argmin R\left( v^{\left( k \right)} \right)+\frac{\mu_{2}}{2}\left\| f^{\left( k \right)}-v^{\left( k \right)}+\frac{\gamma_{2}}{\mu_{2}} \right\|_{2}^{2}$ (S4)

$w^{\left( k+1 \right)}=argmin I_{+}\left( w^{\left( k \right)} \right)+\frac{\mu_{3}}{2}\left\| f^{\left( k \right)}-w^{\left( k \right)}+\frac{\gamma_{3}}{\mu_{3}} \right\|_{2}^{2}$ (S5)

The hyperspectral intensity profiles are in turn updated in each iteration by

$\begin{aligned} f^{\left( k+1 \right)}&=argmin \frac{\mu_{1}}{2}\left\| \mathbf{SC}f^{\left( k \right)}-u^{\left( k+1 \right)}+\frac{\gamma_{1}}{\mu_{1}} \right\|_{2}^{2} \\ &+\frac{\mu_{2}}{2}\left\| f^{\left( k \right)}-v^{\left( k+1 \right)}+\frac{\gamma_{2}}{\mu_{2}} \right\|_{2}^{2}+\frac{\mu_{3}}{2}\left\| f^{\left( k \right)}-w^{\left( k+1 \right)}+\frac{\gamma_{3}}{\mu_{3}} \right\|_{2}^{2} \end{aligned}$ (S6)

where $k$ is the iteration number. The quadratic form of Eqs. (S3) and (S6) determines that they have closed-form solutions. Eq. (S4) is equivalent to a denoising step for $v$ with a prior $R\left( v \right)$. The concept of plug-and-play (PnP) suggests that an off-the-shelf image denoising algorithm can substitute for minimization of Eq. (S4) without specifying $R\left( \cdot\right)$ before running the ADMM [S1]. In our reconstruction, the total variation (TV) [S2], the block-matching and 3D filtering (BM3D) [S3], and the fast and flexible denoising convolutional neural network (FFDNet) [S4] are used as denoisers successively in the whole iteration process. Eq. (S5) represents a projection to a non-negative set and can be carried out by a ramp function.

In each iteration step, the relative difference (i.e. $l_{2}$ norm) of the reconstructed hyperspectral optical intensity profile and its value in the previous step is calculated

$\eta=\frac{\left\| {f^{(k+1)}- f}^{(k)} \right\|_{2}}{\left\| f^{(k+1)} \right\|_{2}}<\rho$ (S7)

Once the calculated relative difference is smaller than a pre-set tolerance value $\rho$, the iteration is terminated. The tolerance value is in the range of 0.001 to 0.01 in our reconstructions.

Finally, we would like to explain how the coded pattern is determined from the experimental data. The compressed sensing based algorithm reconstructs a $N_{x}\times N_{y}\times N_{\omega}$ datacube from a ${(N}_{x}+N_{\omega}-1)\times N_{y}$ two-dimensional signal $A$. To map the spectral $\omega$ information onto the spatial domain $x$, we have pre-calibrated the system with a narrow bandpass filter. When the filter is inserted right before the coded aperture, quasi-monochromatic light corresponding to a specific frequency component is captured by the CCD camera, providing the coded pattern for the specific wavelength. By rotating the orientation of the bandpass filter, the coded patterns for several different wavelengths or frequency components are obtained, and provide calibration information for the CASSI reconstruction. Using this calibration scheme, we can measure the hyperspectral intensity profile with high accuracy. Fig. S1 shows the laser pulse spectrum at a specific transverse position, the CASSI reconstruction (red solid line) well reproduces that independently measured by a fiber-coupled spectrometer (blue dashed line).

**Figure S1. CASSI reconstruction of the spectrum of the incident laser field.** Measured laser spectrum which is reconstructed by CASSI (red line) and as a comparison, the spectrum is independently measured by a fiber spectrometer (blue dashed line).

1. **Single-shot spectral phase measurement using a non-collinear FROG in COFT based on wave front retrieval and non-collinear FROG**

Frequency-resolved optical gating (FROG) can be operated either in a time-scanning mode or a single-shot mode. In the single-shot mode, two copies of the measured laser pulse cross each other at the nonlinear crystal (e.g. a second-harmonic generation SHG crystal) (Fig. S2), and it is assumed that the incident laser pulse has a uniform spatial distribution. Thus, along the transverse p-direction, the two laser pulse copies have different time delays at different positions, mapping the time delay to the transverse spatial position which allows single-shot measurement.

As discussed in the main text, actual laser pulses or optical light fields have different types of spatiotemporal couplings, the assumption that a laser pulse is transversely uniform is no longer available. It is necessary to modify the FROG phase retrieval algorithm by considering the non-uniformity of the field transverse distribution. Thanks to the CASSI measurement, the spatial optical field profile $E_{\omega}\left( x,y \right)=|E_{\omega}\left( x,y \right)|e^{i\varphi_{\mathrm{Spatial}}^{\left( \omega\right)}(x,y)}$ for each frequency component is available, thus only the one-dimensional spectral phase $\varphi_{\mathrm{Spectral}}^{\left( x_{0},y_{0} \right)}(\omega)$ at a specific spatial position is needed to be reconstructed.

**Figure S2. Schematic diagram of non-collinear FROG for single-shot spectral phase measurement.**

We first applied a coordinate transform for the two laser copies from their spatiotemporal coordinates $(x,t)$ to the crystal coordinates $(p,q)$.

$E_{1}\left( p,q \right)=E\left( pcos\theta+qsin\theta,t^{'}-{qcos\theta}/v+{psin\theta}/v \right)$ (S8)

$E_{2}\left( p,q \right)=E\left( pcos\theta-qsin\theta,t^{'}-{qcos\theta}/v-{psin\theta}/v \right)$ (S9)

where $t^{'}$ is real time and $v$ is the light speed in the medium. In the co-moving frame, we define ${t=t}^{'}-{qcos\theta}/v-{psin\theta}/v$, and the FROG signal is

$I_{FROG}\left( p,\omega,\tau\right)={\mathcal{|F}\left[ \int_{0}^{L} E\left( pcos\theta+qsin\theta,t \right)E\left( pcos\theta-qsin\theta,t-\tau\right)dq \right]|}^{2}$ (S10)

Here,$\mathcal{F}$ denotes the Fourier transformation, $\tau={2psin\theta}/v$ is the time delay determined by the transverse position, and $L$ is the medium thickness. In the uniform beam profile case, Eq. (S10) becomes the form of a standard FROG trace $I_{FROG}\left( \omega,\tau\right)={\mathcal{|F}\left[ E(t)E(t-\tau) \right]|}^{2}$.

When the frequency-resolved spatial optical field profile $E_{\omega}\left( x,y \right)$ is available, the standard but complex PCGPA (Principle Components Generalized Projection Algorithm) is not necessary for spectral phase FROG reconstruction. Instead, we have applied an evolutionary algorithm to search for the one-dimensional spectral phase. For the case of our problem, an initial population with members $\varphi_{x_{x_{0},y_{0}}}^{(i)}\left( \omega\right)$ is set up, with $i=1,\ldots,D$, $D$ is the total number of members in the population. Generally, a large population avoids the result stagnation at local minima but requires more computation time due to a slower convergence. To evaluate the fitness of each member in the population, a $G$ value, commonly used to evaluate the convergence in a phase retrieval algorithm, is calculated, with lower values indicating fitter individuals.

$G=\sqrt{\frac{1}{N^{2}}({(I_{meas}\left( \omega,\tau\right)-I_{recon}(\omega,\tau))}^{2}}$ (S11)

We take a Taylor expansion of the spectral phase around the central frequency $\omega_{0}$ that $\varphi_{x_{0},y_{0}}^{(i)}\left( \omega\right)=\sum_{k=0}^{n} \beta_{k}^{\left( i \right)}{\Delta\omega}^{k}=\sum_{k=0}^{n} \beta_{k}^{\left( i \right)}\left( \omega-\omega_{0} \right)^{k}$, and truncates the converging series at $n$ corresponding to negligible dispersions beyond the $n$^th^ order. Thus, the object of searching for the one-dimensional spectral phase is equivalent to reconstructing the dispersion coefficient vector $B=(\beta_{0},\beta_{1},\cdots,\beta_{n})$. For each original member $\varphi^{\left( i \right)}=B^{(i)}W$ in each generation of “mutation” where $W=\left( 1,\Delta\omega,{\Delta\omega}^{2},\cdots,{\Delta\omega}^{n} \right)^{T}$, an offspring is generated through random mutations and crossover [S5]

$B^{\left( o \right)}={P\cdot B}^{\left( i \right)}+\bar{P}\cdot\left[ B^{\left( a \right)}+q\left( B^{\left( b \right)}-B^{\left( c \right)} \right) \right]$ (S12)

$\varphi^{(o)}$=$B^{\left( o \right)}W$ (S13)

where $P$ is an (n+1)-dimensional random Boolean vector and $\bar{P}$ denotes the NOT operation on $P$, $\cdot$ denotes the Hadamard product, and $B^{\left( a \right)}$, $B^{\left( b \right)},$ and $B^{\left( c \right)}$ are randomly selected from the population except $B^{\left( i \right)}$ and arranged by decreasing fitness, and $q$ is a random number between 0 and 1. Once the “offspring” spectral phase is calculated using Eq. (S13), the $G$ values for both all parent $\varphi^{\left( i \right)}$ and offspring $\varphi^{\left( o \right)}$are calculated, and the members with the lowest $G$ values are eliminated from the population in this generation of “evolution”, leaving $D$ members for next generations of evolution until the $G$ values for all population members converge to a small number below tolerance.

To justify the algorithm, we have conducted a simulation reconstructing the spectral phase of a femtosecond laser pulse (Fig. S3(a)) from its non-collinear FROG trace. The pulse has spatiotemporal couplings (i.e. pulse front tilting and curvature), chirp induced temporal broadening, and double-pulse structure (i.e. a post pulse), to challenge the algorithm. Fig. S3(b) shows the reconstructed laser field spatiotemporal structure, demonstrating the capability of the evolutionary algorithm of reconstructing a complex spatiotemporal structure of a laser pulse. In the reconstruction process, we set $n=10$ corresponding to the 10^th^ order of dispersion which is negligibly small. Fig. S3(c) shows the reconstructed spectral phase of interest at a specific transverse location (red dash line), consistent with the originally set spectral phase (blue line). We have also calculated the optimal (blue solid line) and maximum (red dashed line) $G$ factors in each evolution generation (Fig. S3(d)), showing that after evolution generations more than 100 the reconstructed FROG trace fit the measured trace well.

**Figure S3. Simulation results of evolutionary algorithm based FROG. (a)** Spatial-temporal profile of the original pulse. **(b)** Spatial-temporal profile of the reconstructed pulse. **(c)** Spectral phase for the searched location of the original and the reconstructed pulse. **(d)** The evolutionary process of the best and worst individual in the population.

**3. Reconstructing** $\left| \boldsymbol{E(}\boldsymbol{k}_{\boldsymbol{x}}\boldsymbol{,}\boldsymbol{k}_{\boldsymbol{y}}\boldsymbol{,t)} \right|^{\boldsymbol{2}}$ **and** $\left| \boldsymbol{E(x,y,\omega)} \right|^{\boldsymbol{2}}$ **profiles from 3D spectral hologram in COFT based on 3D spectral holography**

As discussed in the main text, CASSI reconstruction algorithm is efficient in recovering the intensity profile of the 3D spectral interference fringes from measured raw data. The intensity profile of the spectral interferogram is expressed in Eq. (2) in the main text. By taking a Fourier transform on $S\left( x,y,\omega\right)$ and separating the direct-current (DC) and alternating-current (AC) terms, the reference pulse amplitude profile $\left| E(x,y,\omega) \right|$ and the signal pulse spectral phase shift $\theta\left( x,y,\omega\right)$ at the Kerr medium are obtained.

Next, we should focus on the signal spectral phase shift $\theta\left( x,y,\omega\right)$, which is supposed to be proportional to the gating spatiotemporal intensity profile at the Kerr medium:

$\theta\left( x,y,\omega\right)\propto\left| E_{pump}\left( x,y,\omega=t/b \right) \right|^{2}=\left| E\left( k_{x}=\frac{x}{a},k_{y}=\frac{y}{a},t=b\omega\right) \right|^{2}$ (S14)

Here $a$ is known and related to the focal length of L1 in Fig. 4(a) in the main text, describing the angular spectra distribution of the incident laser pulse at the far field. The unknown, spatially-resolved parameter $b$ is the linear mapping coefficient linking the chirped signal frequency and the time delay relative to the signal [S6]. Thus, the signal spectral phase shift is equivalent to the intensity profile of the incident laser pulse in the domain of $(k_{x},k_{y},t)$, which is Fourier conjugate to the $\left( x,y,\omega\right)$ domain where we measured the reference amplitude profile.

Here, we apply a pre-estimation method to determine parameter $b$ before conducting three-dimensional phase retrieval. Since the coefficient $b$ is significantly determined by the second-order dispersion coefficient of the reference pulse, with the previously characterized external phase induced by the two glass rods, the averaged reference and gating pulses are estimated by

$E\left( \omega\right)=\left( \int{|E\left( x,y,\omega\right)|}^{2}dxdy \right)^{\frac{1}{2}}e^{i\varphi_{g1}\left( \omega\right)+i\varphi_{g2}\left( \omega\right)+i\beta_{2}\omega^{2}}$ (S15)

$E_{pump}\left( \omega\right)=\left( \int{|E\left( x,y,\omega\right)|}^{2}dxdy \right)^{\frac{1}{2}}e^{i\varphi_{g1}\left( \omega\right)+i\beta_{2}\omega^{2}}$ (S16)

where $\varphi_{gi}\left( \omega\right), i=1, 2$ are the spectral phase induced by pre-calibrated glass rods GR1 and GR2. For each $\beta_{2}$, a unique $b$ can be uniquely determined by the optical field of $E(\omega)$, resulting in an averaged gating pulse profile in the temporal domain

$\left| E_{pump}(t) \right|=\sqrt{\int dxdy\theta\left( x,y,\omega\right)}$ (S17)

After a normalization procedure on $\left| E_{pump}(t) \right|$and $E_{pump}\left( \omega\right)$, we can define a parameter $\Delta S=\int{||E_{pump}\left( t \right)|}^{2}-\left| \mathcal{F}^{-1}\left[ \tilde{E}_{pump}\left( \omega\right) \right] \right|^{2}|dt$ to describe the discrepancy between the temporal profiles of measured and reconstructed phase shift. Fig. S4(a) shows that $\Delta S$ reaches the minimum when $\beta_{2}=80$ fs^2^, corresponding to $b=8075$ fs^2^.

Note that the pre-estimation step neglects the spatial difference of the parameter $b$ and is valid only when the initial group velocity dispersion $\beta_{2}$ of the incident laser field is significantly smaller than the glass rods induced chirp $\varphi_{gi}\left( \omega\right), i=1, 2$ that the time-frequency mapping parameter $b$ is predominantly determined by the spatially-uniform chirp phase $\varphi_{gi}\left( \omega\right)$ induced by the glass rods. In our experiment (Fig. 4 in the main text), the incident optical field is a well-compressed laser pulse from a commercial femtosecond laser amplifier with negligible second-order chirp $\beta_{2}$ compared to the glass rods. Using the reconstructed three-dimensional optical field (Fig. 4(b) in the main text), we can calculate the spatial distribution of the parameter $b$ using the reconstructed spatially-resolved $\beta_{2}$ [Fig. S4(b)], and the maximum variation of less than 5% is negligible.

**Figure S4. (a)** The $\beta_{2}$ determined parameter $b$ and $\Delta S$. **(b)** The spatial distribution of $b$ after finishing the three-dimensional phase retrieval.

For optical field with significant initial chirped or spatiotemporal coupling, a modified three-dimensional phase retrieval algorithm, with the spatially resolved $b$ estimation embedded in the phase retrieval iterations, should be developed. Starting with the initial guess of $b$ through the pre-estimation step and in each iteration, the temporal profile of phase shift $\theta\left( x,y,t \right)$ can be determined with the knowledge of $\theta\left( x,y,\omega\right)$ and $b(x,y)$. So the three-dimensional optical field distribution is obtained and $b(x,y)$ is in turn updated with $\beta_{2}(x,y)$ information. Such iteration loops continue until the reconstructed optical field and $b(x,y)$ converge.

**Reference**

S1. Venkatakrishnan, S., Bouman, C. A. & Wohlberg, B. Plug-and-Play Priors for Model Based Reconstruction. *2013 IEEE Global Conf. Signal Inf. Process.* 945-948 (2013).

S2. Chambolle, A. An algorithm for total variation minimization and applications. *J. Math. Imaging Vis.* **20**, 89-97 (2004).

S3. Dabov, K., Foi, A., Katkovnik, V. *et al*. Image denoising by sparse 3-D transform-domain collaborative filtering. *IEEE Trans. Image Process.* **16**, 2080-95 (2007).

S4. Zhang, K., Zuo, W. & Zhang, L. FFDNet: Toward a Fast and Flexible Solution for CNN based Image Denoising. *IEEE Trans. Image Process.* **27**, 4608-4622 (2018).

S5. Escoto, E., Tajalli, A., Nagy, T. *et al*. Advanced phase retrieval for dispersion scan: a comparative study. *J. Opt. Soc. Am. B* **35**, 8-19 (2018).

S6. Kim, K. Y., Alexeev, I. & Milchberg, H. M. Single-shot supercontinuum spectral interferometry. *Appl. Phys. Lett.* **81**, 4124-4126 (2002).
